# Can prophylactic breast irradiation contribute to cardiac toxicity in patients with prostate cancer receiving androgen suppressing drugs?

**DOI:** 10.1186/1748-717X-3-2

**Published:** 2008-01-10

**Authors:** Carsten Nieder, Adam Pawinski, Nicolaus H Andratschke, Michael Molls

**Affiliations:** 1 Radiation Oncology Unit, Nordlandssykehuset HF, 8092 Bodø, Norway; 2 Department of Radiation Oncology, Klinikum rechts der Isar der Technischen Universität München, 81675 Munich, Germany

## Abstract

**Background:**

Androgen suppression treatment (AST) might increase the risk of cardiac morbidity in prostate cancer patients. Possible explanations were provided, however, they disregard the potential contribution of prophylactic radiotherapy to the mamillary regions (PMRT, prescribed to avoid gynecomastia).

**Methods:**

We studied the exposure of the heart in a typical electron beam PMRT setting by evaluating computed tomography (CT) scans in 40 non-cancer patients (age 65 and 75 years in 50% each) and 17 prostate cancer patients. Five of the younger, 7 of the older and 4 of the cancer patients had significant cardiac disease.

**Results:**

The median distance between skin and outer heart contour decreased with age. In all three groups, patients with cardiac morbidity had smaller distances. When using the CT-determined PMRT beam energy, 10% of the younger, 15% of the older and none of the prostate cancer patients would receive approximately 50% of the prescription dose to a part of the heart (2 had no history of cardiac disease). When using the clinically rather than CT-determined beam energy, as often done in daily practice, an additional 12.5% of the non-cancer and 12% of the prostate cancer patients would be exposed to comparably high doses.

**Conclusion:**

The present data provide preliminary evidence that PMRT might be a factor that contributes to cardiac side effects. Previous studies that established a relationship between AST and cardiac morbidity did not include information on delivery of PMRT.

## Background

Androgen suppression including temporary suppression in patients receiving curative radiation therapy represents an important treatment option for patients with prostate cancer [1]. One of the disadvantages and side effects of androgen suppression is the increased risk of cardiac toxicity, another one the risk of gynecomastia development [2-4], e.g., during treatment with goserelin acetate and flutamide [5] or with bicalutamide [6-8]. Prophylactic radiation therapy to both mamillar regions (PMRT) before the start of androgen suppression might decrease the likelihood of gynecomastia [7,9,10]. However, depending on the anatomical situation, left-sided PMRT might lead to a certain exposure of the heart to ionizing radiation.

Typically, single electron beams with a sharp dose gradient are used, having the advantage of limited tissue penetration. In contrast to most other situations in contemporary radiation oncology, no 3-dimensional computed tomography (CT)-based treatment planning is used. Therefore, the exact dose distribution is unknown for the individual patient, leaving room for accidental dose exposure of the heart. In the health region of Northern-Norway for example, where one of the authors' institutions is located, a standard clinical set-up for PMRT is used. It consists of a single dose of 15 Gy delivered via circular fields, diameter 7 cm, electron energy 9 MeV (6 and 12 MeV in slim and obese patients, respectively). Both the left and right perimamillar regions are treated with one such field. Using similar techniques, the authors from Munich, Germany, administer 3 fractions of 4 Gy each. Both regimens are among those previously studied by different groups, where PMRT was found to prevent gynecomastia development [7,9,10].

Recent articles provide possible explanations for the elevated risk of cardiac diseases in patients treated with androgen suppression, e.g., changes in lipid metabolism [11]. However, we hypothesised that administration of PMRT might further contribute to long-term toxicity in a multifactorial scenario. Therefore, the present study examined potential radiation doses to the heart in a group of 40 individuals who underwent thoracic imaging for various medical reasons and 17 patients with prostate cancer.

## Methods

We first analysed 40 male patients who received contrast-enhanced CT scans of the thorax for various medical reasons (unrelated to cancer treatment) after appropriate institutional informed consent. Twenty patients were 65 years old and 20 were 75 years old. They were selected from the radiology departments database (Nordlandssykehuset, Bodø, Norway) based on their date of birth. The search was started with patients born 01. June 1942 and 1932, respectively, and continued towards the end of the year until 20 patients were identified in each group. They were not allowed to have significant lung abnormalities such as previous surgery, tumors or pleural effusions. All medical records were also available in the hospital's data system. They were reviewed to identify those patients with a history of serious heart disease such as myocardial infarction, aortocoronar bypass surgery and other coronary artery interventions. Asymptomatic coronary artery disease, elevated blood pressure or mild types of cardiac dysfunction were not considered for the purpose of this study.

**Figure 1:**
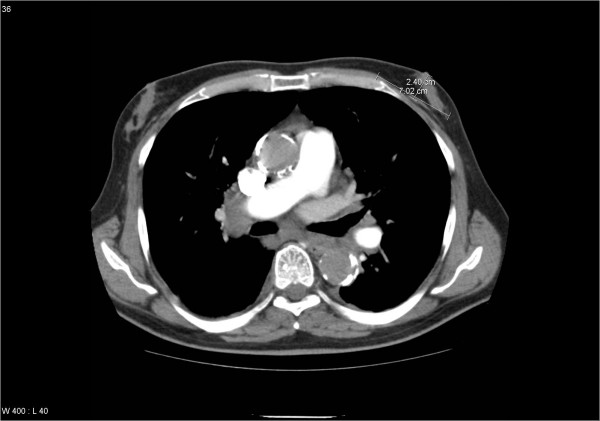
Axial contrast-enhanced computed tomography scan at the level of the left mamilla displaying both the distance between the skin surface and the pectoral musculature (2.4 cm) and the field size of 7 cm. Note that only very low heart exposure results from electron beam irradiation at this level, i.e. the center of the field.

In each patient, the left mamilla (center of the PMRT field) was identified on the CT scans and the distance between the skin and anterior border of the pectoral musculature was measured (Figure 1). This value was used to calculate the electron beam energy needed for PMRT. Previously published electron depth-dose distribution data (Table 1) were used. The therapeutic depth of the electrons was to match the anterior border of the pectoral musculature, which corresponds to the posterior border of the target volume, as closely as possible. Then, both the optimal CT-based electron beam and the clinically used standard 9 MeV beam were chosen for further evaluation. At a caudal distance of 3 cm from the mamilla, i.e. close to the inferior field border, the dose to the heart was estimated. As evident from the CT scans, only the distal parts of the field might cause relevant doses to the heart. We measured the distance between the skin and the outer contour of the heart and used the data from Table 1 to estimate the heart dose. The same methods were used to examine the first 17 patients with prostate cancer who were treated since the opening of the Radiation Oncology Unit at Nordlandssykehuset in June 2007. Not all of these patients actually received PMRT, some were treated for metastatic disease. Finally, the CT scans of the prostate cancer patients, which were available in our treatment planning system (Varian Eclipse), were used to calculate actual 3-D dose distributions and dose-volume histograms in representative cases, i.e. those patients where the left anterior descending coronary artery (LAD) could be identified. Varian Eclipse uses the Generalized Gaussian Pencil Beam algorithm for calculating electron dose distributions. The plans were calculated for a Varian Clinac treatment unit.

**Table 1:** Electron beam dose distribution (values might vary, e.g., with field size, source-skin-distance and tissue homogeneity), adapted from [22].

Beam energy	Surface dose	Therapeutic depth	Depth of 50% isodose
6 MeV	72%	20 mm	24 mm
9 MeV	78%	30 mm	38 mm
12 MeV	83%	40 mm	50 mm

## Results

Out of 20 65-years-old patients, 5 had a history of significant cardiac disease. In the 75-years-old group, 7 patients belonged to this subset. Among the prostate cancer patients, 4/17 had significant cardiac disease. The latter group had a median age of 72 years, range 58–83 years. The required beam energy for PMRT was different from 9 MeV in the majority of patients. While 6 patients in both non-cancer-groups actually were best treated with 9 MeV, 11 and 13 patients in the 65-years and 75-years group would have benefited from choosing 6 MeV. In 3 and 1 individuals, 12 MeV were necessary to cover the pre-pectoral region adequately. In the prostate cancer patients, 9 MeV was appropriate in 6 cases, 6 MeV in 8 cases, 12 MeV in 2 cases and 15 MeV in 1 case.

**Figure 2:**
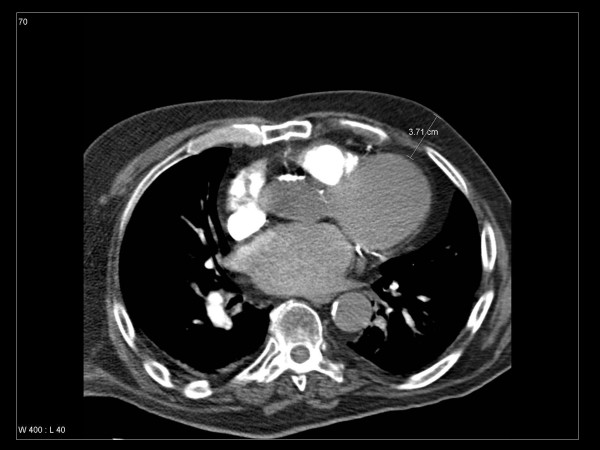
Axial contrast-enhanced computed tomography (CT) scan 3 cm caudal from the mamilla displaying the approximate depth of the 50% isodose from the CT-determined 9 MeV electron beam. In this 65-years-old non-cancer patient with previous heart disease, parts of the left ventricle would be exposed to unexpected doses of ionizing radiation.

**Figure 3:**
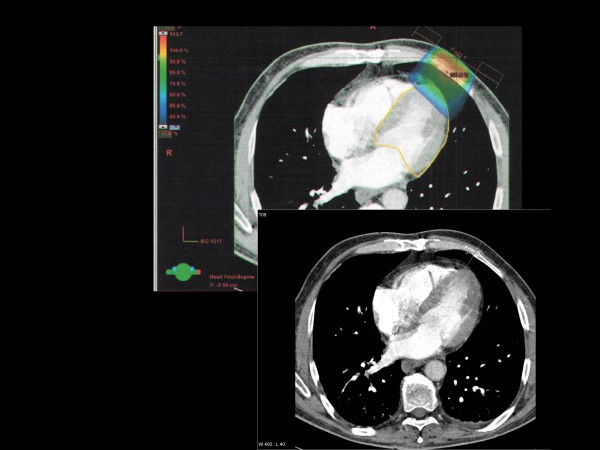
Axial contrast-enhanced computed tomography scan 3 cm caudal from the mamilla displaying on the lower image the approximate depth of the 50% isodose from a standard 9 MeV electron beam (6 MeV would have been appropriate). 3-D planning illustrates that the actual dose to the heart is even higher. The left ventricle (contoured in yellow) is the part of the heart that receives the highest dose (maximum 80%). The blue isodose wash refers to 33% of the prescription dose, i.e. 5 Gy.

The median distance between skin and outer heart contour decreased with age from 6.25 cm in the 65-years group to 5.35 cm in the 75-years group (range 3.1–8.7 cm and 2.6–8.7 cm, respectively). In prostate cancer patients, 5.5 cm were measured (range 3.8–8.1 cm). In all three groups, patients with cardiac morbidity had smaller distances. In the 65-years-old patients, the median values were 5.1 vs. 6.7 cm for patients with/without serious heart disease. In the older patients these figures were 4.2 vs. 5.6 cm. In the prostate cancer patients, 4.8 vs. 5.7 cm were calculated. For all groups combined, 5.0 vs. 6.4 cm were calculated. When using the CT-based beam energy, two of the younger non-cancer patients (10%) would receive ≥50% of the prescription dose to a relatively small part of the anterior myocardial wall of the left ventricle and the small vessels in this region. Both patients had a history of cardiac disease (Table 2). Among the older patients, one would receive ≥50% to a small heart volume, while two would receive ≥50% to a more extended part of the heart (total 5/40 patients, 12.5%). Only one of these three 75-years-old patients had a history of cardiac disease (Figure 2). None of the prostate cancer patients would receive comparably high doses to the heart when CT-based beams were used. When using the inappropriate 9 MeV beam rather than the optimal 6 MeV beam, one additional younger non-cancer patient plus four additional older patients would receive an unnecessary heart exposure. In the absence of CT information, two of the prostate cancer patients (12%) would belong to the group with unnecessary heart exposure when using the 9 MeV beam rather then the optimal 6 MeV beam (Figure 3). The use of the 12 or 15 MeV beam, where appropriate in obese patients would be possible without concerns.

**Table 2:** Individual data of patients with heart exposure from prophylactic breast radiation therapy.

Patientnr.	Age (years)	Heart disease	CT-based beam energy	Skin-heart distance	Exposure
1	65	yes	12 MeV	5,1 cm	Moderate
2	65	yes	9 MeV	4,2 cm	Moderate
3	75	yes	9 MeV	4,0 cm	Distinct
4	75	no	6 MeV	2,6 cm	Moderate
5	75	no	9 MeV	3,7 cm	Distinct
6	65	no	6 MeV	3,1 cm	Moderate**
7	75	no	6 MeV	3,8 cm	Moderate**
8	75	yes	6 MeV	4,0 cm	Moderate**
9	75	yes	6 MeV	3,9 cm	Moderate**
10	75	yes	6 MeV	3,3 cm	Distinct**
11*	64	no	6 MeV	3,9 cm	Moderate**
12*	83	no	6 MeV	3,9 cm	Moderate**

* prostate cancer patient

**when using the standard 9 MeV beam in the absence of CT scan information

The 3-D dose distributions were first evaluated in prostate cancer patients for the 9 MeV beam, even though this energy would not be appropriate if CT information was available for treatment planning. The examples revealed that the mean dose to the heart is in the range of 2 to 5% of the prescription dose. Five percent corresponds to 0.75 Gy if one uses a single fraction of 15 Gy. The proximal parts of the LAD received up to 14% of the prescription dose, i.e. 2.1 Gy. The distal parts were indistinguishable from the myocardium of the left ventricle with the CT protocols used in these patients. In general, the highest doses to the heart were seen in the anterior part of the left ventricle and the interventricular septum (Figure 3). Up to 80% of the prescription dose was observed in very small volumes (<3%) of these areas. Even the volume of the left ventricle receiving 50% of the dose, i.e. 7.5 Gy, was comparably small (maximum 5%). Up to 10% of the left ventricle received 25% of the dose, i.e. 3.75 Gy, and up to 18% received 10% of the dose, i.e. 1.5 Gy. If one takes the patients' individual anatomy into account and selects the 6 MeV beam in such cases, the doses to the left ventricle decrease drastically. The same small volumes that would receive 50–80% of the dose with the 9 MeV beam, would so receive 10–20% and the mean dose to the left ventricle would not exceed 5% of the prescription dose, i.e. 0.75 Gy.

## Discussion

The present analysis is to our knowledge the first one that addresses the role of PMRT as a potential cause of cardiac morbidity in prostate cancer patients receiving androgen suppression therapy. It was performed both in cancer patients and randomly selected individuals having had CT examinations for other medical reasons. The results in these groups were largely comparable. We used 3-D treatment planning with display of isodose distributions and dose-volume histograms only in those patients whose CT scans already were entered into the treatment planning system, i.e. prostate cancer patients, and only if the LAD could be identified. Data from these patients suggest that parts of the left ventricle might be exposed to 50–80% of the prescription dose, even if the mean doses in general are low. Studies in electron boost treatment for breast cancer have also shown that the heart might be exposed to unexpected radiation doses in a proportion of these patients [12]. The present data suggest that standard non-CT-based approaches often are unsatisfactory and that individual 3-D treatment planning might benefit a considerable number of patients because it can reduce the radiation dose to the heart. This benefit appears to increase with patient age and pre-existing cardiac morbidity. Even among those treated with the appropriate beam energy, up to 12.5% of the patients might be at risk for exposure of the heart to unnecessary radiation doses. This figure increases when the beam energy is determined just on the basis of a clinical examination without exact anatomical information.

We arbitrarily decided to depict in Figure 2 the depth where approximately 50% of the prescription dose is administered. At first glance, 50% of a prescription dose of 15 Gy (single fraction) or 12 Gy (in 3 fractions) appears relatively low compared to the heart doses reported from radiation treatment in a variety of mediastinal tumors [13]. Several data sets suggest, however, that doses as low as 4–5 Gy might contribute to cardiac toxicity [14-16]. These epidemiologic findings are largely compatible with radiobiologic data on the pathogenesis of radiation-induced heart disease, as comprehensively reviewed by Schultz-Hector and Trott [17]. The endothelial lining of blood vessels might be particularly vulnerable, resulting in slowly progressive functional and structural alterations. On the basis of these findings, even partial heart exposures might contribute to long-term damage, which typically becomes manifest after several years [18]. In reality, the 50% isodose might reach even further into the heart than displayed in Figure 2, because the air-containing lungs allow for deeper penetration of the electron beam than soft tissues. Figure 3 confirms that the 50% isodose depth taken from the values in Table 1 might underestimate the actual dose distribution in a patient.

Is it possible to relate or fit our preliminary findings to the published cardiac toxicity data? An observational study of a population-based cohort of 73,196 Medicare enrollees age 66 years or older who were diagnosed with locoregional prostate cancer during 1992 to 1999 and observed through 2001 was recently published [2]. The authors analysed in this Surveillance, Epidemiology and End Results database whether treatment with GnRH agonists was associated with coronary heart disease, myocardial infarction, and sudden cardiac death. Men with prevalent diabetes and coronary heart disease were excluded. The mean age at diagnosis was 74 years. More than one third of men received a GnRH agonist during follow-up. GnRH agonist use was associated with increased risk of coronary heart disease (adjusted HR, 1.16; P < .001), myocardial infarction (adjusted HR, 1.11; P = .03), and sudden cardiac death (adjusted HR, 1.16; P = .004). Therapy for as few as 1–4 months was associated with an increased risk of coronary artery disease. Unfortunately, the database did not include information about use of oral antiandrogens, combined androgen blockade and PMRT in this cohort.

Another group evaluated whether the timing of fatal myocardial infarction was influenced by the administration of androgen suppression therapy [3]. The study cohort comprised 1,372 men who were enrolled onto three randomized trials between 1995 and 2001. In the three trials, the men were randomly assigned to receive radiation therapy with 0 versus 3 versus 6, 3 versus 8, or 0 versus 6 months of androgen suppression (goserelin plus flutamide or a GnRH agonist only). The median age was 68–72.5 years in the three trials. Men age 65 years or older who received 6 months of androgen suppression experienced shorter times to fatal infarction compared with men in this age group who did not receive such medication (P = .017). Even three months of treatment might shorten the time to fatal myocardial infarction, but additional evidence is needed to strengthen this hypothesis. As communicated by the principal investigators, PMRT was not offered in two of the trials, while the exact proportion of patients that received this treatment is unknown from the Canadian trial (personal communication, July 2007). It is therefore not possible to compare the available clinical results with the percentage of patients that might receive relevant radiation doses to the heart in our present study. Importantly, other data from patients treated with radiation therapy plus androgen suppression also suggest that hormonal manipulation might result in greater non-cancer mortality [19].

Despite the fact that a causal relationship between the relatively low radiation doses from PMRT and cardiac morbidity or mortality can not be proven at this time, it appears prudent to minimize all factors that might contribute to non-cancer mortality in these patients. Even if PMRT should be considered as just one of the potential factors contributing to cardiac morbidity in patients receiving androgen suppression therapy, the question arises whether the use of non-3-dimensional planning and treatment techniques should continue in an era where advanced technology that reduces the dose to the heart and takes, e.g., advantage of breathing control, which might help to increase the distance between thoracic wall and heart, is available [20] and where the occasional patients with still unacceptable radiation treatment plans can switch to alternative treatments such as tamoxifen [7]. In addition, androgen suppression regimens with lower rates of symptomatic gynecomastia might be considered [21]. Future epidemiologic studies on cardiac side effects of androgen suppression should try to include data on the use of PMRT [Additional file 1].

## Conclusion

The present data provide preliminary evidence that PMRT might be a factor that contributes to the cardiac side effects of androgen suppression therapy in certain patients where the distance between the PMRT target volume and the outer heart contour is small. Previous studies that established a relationship between androgen suppression and cardiac morbidity did not include information on delivery of PMRT in their patient cohorts.

## Competing interests

The author(s) declare that they have no competing interests.

## Authors' contributions

CN and AP carried out the data acquisition and analysis. CN and NHA drafted the manuscript. CN, NHA and MM participated in the design of the study. All authors read and approved the final manuscript.

## Supplementary Material

Additional file 1 Correspondence published in the Journal of the National Cancer Institute. The text provided represents a recent publication on the same topic. Click here for file
