# Supplementary material for: Can prophylactic breast irradiation contribute to cardiac toxicity in patients with prostate cancer receiving androgen suppressing drugs?
Source: Radiat Oncol. 2008 Jan 10;3:2. doi: 10.1186/1748-717X-3-2 (PMC2249590; doi:10.1186/1748-717X-3-2)
Supplement: Additional file 1 — Correspondence published in the Journal of the National Cancer Institute. The text provided represents a recent publication on the same topic. [file 1748-717X-3-2-S1.pdf]

JNCI	djm188	LM
JOURNAL NAME	MS NO	CE Code

# CORRESPONDENCE

Does Prophylactic Breast Irradiation in Patients With Prostate Cancer Influence Cardiac Toxicity?

Androgen suppression, including temporary suppression in patients who receive curative radiotherapy, is often used for patients with prostate cancer, but it might lead to cardiac toxicity and gynecomastia (1–3). Prophylactic radiation therapy to [AQ1] both mamillar regions (PMRT••) before the start of androgen suppression might decrease the likelihood of gynecomastia (4–6). In practice, many centers use single-electron beams with a sharp dose gradient without three-dimensional computed tomography (CT)–based treatment planning. However, in some patients, left-sided PMRT might lead to exposure of the heart to ionizing radiation.

We analyzed CT scans from 40 male patients who were 65 or 75 years old. Each patient's left mamilla (center of the PMRT field) was identified on the CT image, and the distance between skin and anterior border of the pectoral musculature was measured and used to calculate the electron beam energy needed for PMRT. We then evaluated the irradiation dose to the heart using the optimal CT-based electron beam energy and a clinically determined beam energy. The standard clinically determined beam is a 9-MeV electron beam, which covers a tissue depth of approximately 3 cm from the skin surface. In large ••patients, 12 MeV is used and in thin patients, 6 MeV. The latter cover 4 and 2 cm, respectively••.

The median distance between skin and outer heart contour decreased with age from 6.25 to 5.35 cm. In both age groups, patients with known serious cardiac morbidity had shorter distances than patients of the same age without cardiac morbidity. When using the CT-based beam, 10% of the younger patients and 15% of the older patients would receive approximately 50% of the prescription dose to a part of the anterior myocardial wall of the left ventricle and the small vessels in this region (Fig. 1). [F1] When using the clinically determined beam, which is often used in practice, an additional 12.5% of the patients would be exposed to a dose comparable to that from the CT-based beam.

This preliminary analysis that was performed in randomly selected individuals who had CT examinations for various medical reasons suggests that CT-based approaches [AQ2] for PMRT might benefit approximately 20%–28% of patients because it can reduce [AQ3] the radiation dose to the heart compared with non-CT–based approaches. Data suggest that doses as low as 4–5 Gy might contribute to cardiac toxicity and are largely in agreement with radiobiologic data on the pathogenesis of radiation-induced heart disease (7). The endothelial lining of blood vessels might be particularly vulnerable to radiation; exposure may result in slow progressive functional and structural alterations. Even partial exposure of the heart to radiation might contribute to long-term damage after several years. Despite the fact that a causal relationship between the relatively low radiation doses from PMRT and cardiac morbidity or mortality has not been proven, it is prudent to minimize all factors that might contribute to noncancer mortality in these patients. Future epidemiologic studies on the cardiac side effects of androgen suppression should include data on the use of PMRT.

CARSTEN NIEDER
ADAM PAWINSKI
NICOLAUS H ANDRATSCHKE
MICHAEL MOLLS

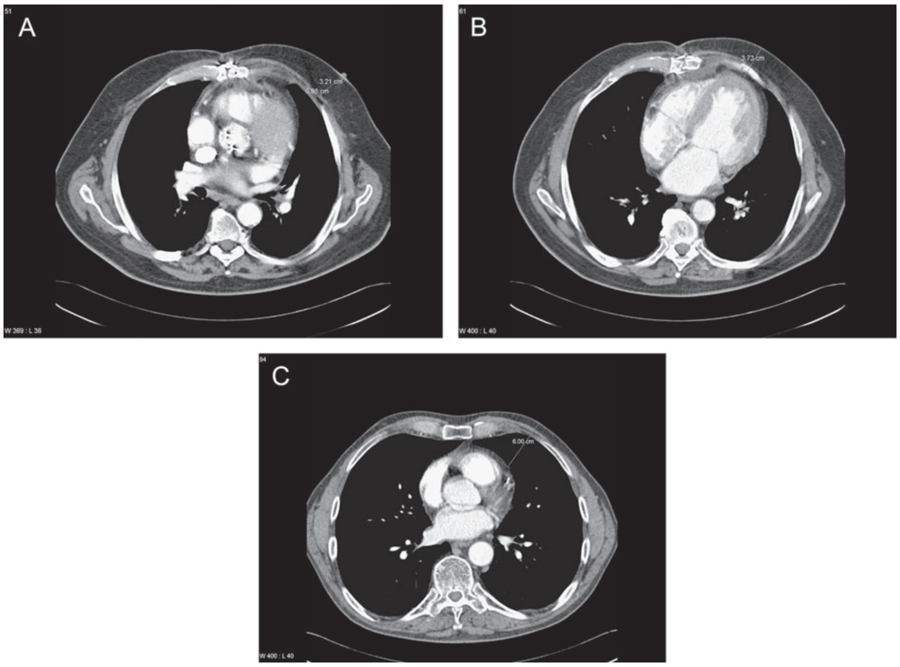

Fig 1. Axial contrast-enhanced computed tomography (CT) scans at the level of the left mamilla (center of the electron field). **A**) A CT scan of a 75-year-old patient with history of cardiac surgery. The distance between the skin surface and the pectoral musculature (3.21 cm) and the standard field size of 7 cm used at Nordlandssykehuset, Bodø, Norway, are shown. **B**) The same patient's scan at a caudal distance of 3 cm from the field center is displayed. Note the exposure to the heart in the medial part of the field, resulting from electron beam irradiation at this level. The distance of 3.73 cm indicated on the scan corresponds to approximately 50% of the prescription dose when a 9-MeV electron beam is used. This beam energy is appropriate in this situation when the distance between skin surface and pectoral muscles is approximately 3 cm. **C**) A CT scan of a 65-year-old patient without cardiac morbidity. Here, the distance between skin and heart is much larger (6 cm). Therefore, the dose to the heart will be very low, even in the caudal and medial parts of the irradiated field.

References

(1) Keating NL, O'Malley AJ, Smith MR. Diabetes and cardiovascular disease during androgen deprivation therapy for prostate cancer. J Clin Oncol 2006;24:4448–56.

(2) D'Amico AV, Denham JW, Crook J, Chen MH, Goldhaber SZ, Lamb DS, et al. Influence of androgen suppression therapy for prostate cancer on the frequency and timing of fatal myocardial infarctions. J Clin Oncol 2007; 25:2420–5.

(3) Higano CS. Side effects of androgen deprivation therapy: monitoring and minimizing toxicity. Urology 2003;61Suppl 1:32–8.
(4) Perdona S, Autorino R, De Placido S, D'Armiento M, Gallo A, Damiano R, et al. Efficacy of tamoxifen and radiotherapy for prevention and treatment of gynaecomastia and breast pain caused by bicalutamide in prostate cancer: a randomised controlled trial. Lancet Oncol 2005;6:295–300.
(5) Tyrrell CJ, Payne H, Tammela TL, Bakke A, Lodding P, Goedhals L, et al. Prophylactic breast irradiation with a single dose of electron beam radiotherapy (10 Gy) significantly reduces the incidence of bicalutamide-induced gynecomastia. Int J Radiat Oncol Biol Phys 2004;60:476–83.
(6) Widmark A, Fosså SD, Lundmo P, Damber JE, Vaage S, Damber L, et al. Does prophylactic breast irradiation prevent antiandrogen-induced gynecomastia? Evaluation of 253 patients in the randomized Scandinavian trial SPCG-7/SFUO-3. Urology 2003;61:145–51.
(7) Schultz-Hector S, Trott KR. Radiation-induced cardiovascular diseases: is the epidemiologic evidence compatible with the radiobiologic data?. Int J Radiat Oncol Biol Phys 2007;67:10–18.

Notes

[AQ4] **Affiliations of authors**: Radiation Oncology Unit, Nordlandssykehuset HF, Bodø, Norway (CN, AP); Department of Radiation Oncology, Klinikum rechts der Isar der Technischen Universität München, Munich, Germany (NHA, MM).

Correspondence to: Carsten Nieder, MD, Department of Internal Medicine, Radiation Oncology Unit, Nordlandssykehuset HF, 8092 Bodø, Norway (e-mail: carsten.nieder@nlsh.no).

DOI: 10.1093/jnci/djm188

© The Author 2007. Published by Oxford University Press. All rights reserved. For Permissions, please e-mail: journals.permissions@oxfordjournals.org.

AUTHOR QUERIES

[AQ1] Au: Should “PMRT” be defined here? Also, please check whether the usage “mamillar” is correct or whether it can be changed to “mamillary.”

[AQ2] Au: SE: Is the usage “large” okay?

[AQ3] Au: Au: edits ok?

[AQ4] Au: Can “Department of Internal Medicine” be inserted before “Radiation Oncology Unit” in the affiliation field?
